# Primary care blood tests show lipid profile changes in pre-symptomatic amyotrophic lateral sclerosis

**DOI:** 10.1093/braincomms/fcad211

**Published:** 2023-07-28

**Authors:** Alexander G Thompson, Rachael Marsden, Kevin Talbot, Martin R Turner

**Affiliations:** Nuffield Department of Clinical Neurosciences, University of Oxford, Oxford OX3 9DU, UK; Nuffield Department of Clinical Neurosciences, University of Oxford, Oxford OX3 9DU, UK; Nuffield Department of Clinical Neurosciences, University of Oxford, Oxford OX3 9DU, UK; Nuffield Department of Clinical Neurosciences, University of Oxford, Oxford OX3 9DU, UK

**Keywords:** amyotrophic lateral sclerosis, motor neurone disease, biomarker, pre-symptomatic, cholesterol

## Abstract

Multiple sources of evidence suggest that changes in metabolism may precede the onset of motor symptoms in amyotrophic lateral sclerosis. This study aimed to seek evidence for alterations in the levels of blood indices collected routinely in the primary care setting prior to the onset of motor symptoms in amyotrophic lateral sclerosis. Premorbid data, measured as part of routine health screening, for total cholesterol, high-density and low-density lipoprotein cholesterol, triglyceride, glycated haemoglobin A1c and creatinine were collected retrospectively from (i) a cohort of amyotrophic lateral sclerosis patients attending a specialist clinic (*n* = 143) and (ii) from primary care–linked data within UK Biobank. Data were fitted using linear mixed effects models with linear b-splines to identify inflection points, controlling for age and sex. In specialist amyotrophic lateral sclerosis clinic cases, models indicated decreasing levels of total and low-density lipoprotein cholesterol prior to an inflection point in the years before symptom onset (total cholesterol 3.25 years, low-density lipoprotein cholesterol 1.25 years), after which they stabilized or rose. A similar pattern was observed in amyotrophic lateral sclerosis cases within UK Biobank, occurring several years prior to diagnosis (total cholesterol 7 years, low-density lipoprotein cholesterol 7.25 years), differing significantly from matched controls. High-density lipoprotein cholesterol followed a similar pattern but was less robust to sensitivity analyses. Levels of triglyceride remained stable throughout. Glycated haemoglobin temporal profiles were not consistent between the clinic and biobank cohorts. Creatinine level trajectories prior to amyotrophic lateral sclerosis did not differ significantly from controls but decreased significantly in the symptomatic period after an inflection point of 0.25 years after symptom onset (clinic cohort) or 0.5 years before diagnosis (UK Biobank). These data provide further evidence for a pre-symptomatic period of dynamic metabolic change in amyotrophic lateral sclerosis, consistently associated with alterations in blood cholesterols. Such changes may ultimately contribute to biomarkers applicable to population screening and for pathways guiding the targeting of preventative therapy.

## Teaser

Using routine health screening blood test data in primary care, Thompson *et al.* demonstrate distinct pre-symptomatic biphasic blood cholesterol trajectories in those who went on to develop amyotrophic lateral sclerosis, implicating metabolic alterations associated with pathogenesis.

## Graphical Abstract

**Graphical Abstract:**
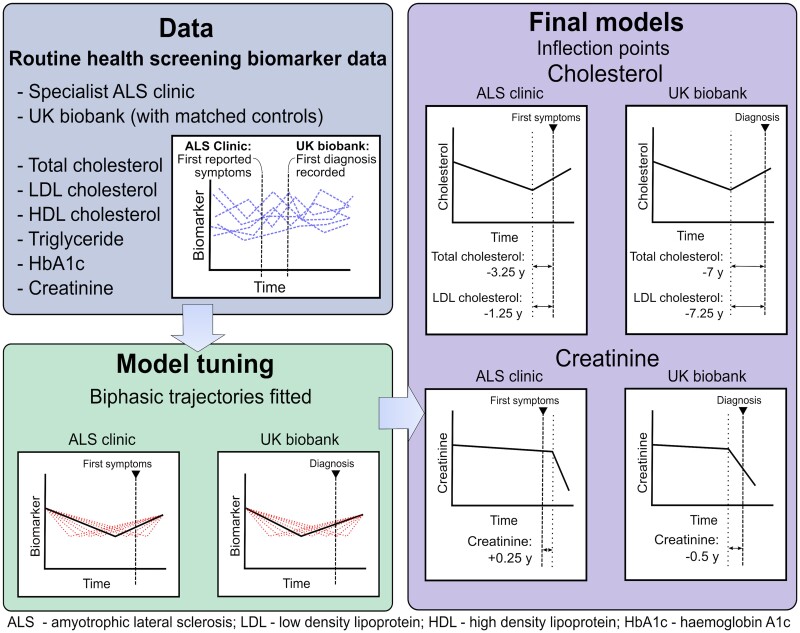

## Introduction

Amyotrophic lateral sclerosis (ALS) is an adult-onset neurodegenerative disease in which the onset of motor symptoms often appears abruptly and follows an unrelenting course associated with an average survival of 2–3 years from initial weakness.^1^ Approximately 10% of cases are attributable to highly penetrant single-gene variants, associated with ALS that is clinically indistinguishable from apparently sporadic disease and shares the pathological hallmark of cytoplasmic TDP-43 inclusions. An apparently precipitous decline of function may follow the exhaustion of compensatory mechanisms and inherent system redundancy. A range of upstream biological changes, converging on a final common pathway, is consistent with epidemiological analysis supporting a ‘multiple hit’ model of pathogenesis.^2,3^ Age is a consistent and major risk factor for ALS and other neurodegenerative disorders. Identifying markers that can predict the onset of neurodegeneration prior to the clinical horizon will be essential to enable therapy before irrecoverable motor neuron loss occurs.

Identifying pre-symptomatic alterations in a relatively rare disorder such as ALS has necessarily focused on people carrying penetrant monogenetic pathological variants. The most sensitive marker of active pathology associated with neuronal loss in gene carriers is elevation in neurofilament proteins in blood and cerebrospinal fluid, but these only rise significantly in the months prior to symptom onset.^4,5^ Significant elevation of the cerebrospinal fluid microglial protein chitotriosidase-1 similarly occurs around the time of symptom onset.^6,7^ Unbiased analysis of the cerebrospinal fluid proteome of asymptomatic ALS gene carriers is yet to identify a consistent set of biochemical pathway alterations in the years prior to expected symptom onset.^8^

Several studies have examined pre-symptomatic blood biochemistry in large cohorts that included people who have gone on to develop apparently sporadic ALS. Neurofilament proteins in blood followed a similar pattern to that seen in ALS gene carriers.^9^ In studies of analytes collected routinely as part of health screening for primary and secondary prevention of cardiovascular disease, higher levels of low-density lipoprotein (LDL) cholesterol and apolipoprotein B^10^ and lower levels of high-density lipoprotein (HDL) cholesterol and apolipoprotein A1^11,12^ were associated with higher risk of later ALS. Some studies of lipid markers have also suggested dynamic changes in levels of LDL and HDL cholesterol in the years prior to the onset of ALS symptoms.^10,11^ Genetic epidemiological analysis has also indicated a causal relationship between higher levels of blood LDL cholesterol levels and ALS.^13^ As well as increasing risk of developing ALS, higher levels of LDL cholesterol and triglycerides have been associated with longer survival in ALS patients,^14-16^ though somewhat inconsistently, with a recent meta-analysis finding no clear association between lipid levels and survival and other work suggesting that survival associations of blood lipids might be confounded by effects of body mass index or related to respiratory dysfunction.^17-19^

Levels of creatinine (a reflection of both muscle mass and renal function) have been shown to diverge from control levels prior to ALS diagnosis.^20,21^ Elevation in the muscle enzyme creatine kinase, which has been inconsistently linked with longer survival in ALS, has also been observed pre-symptomatically.^22,23^

We conducted a retrospective analysis of historical blood test data available in a cohort of patients attending a tertiary ALS clinic including samples obtained in the years prior to their diagnosis. Similar data were examined from participants enroled into a large UK population cohort study (UK Biobank), some of whom went on to be diagnosed with ALS, compared with age- and sex-matched controls. The aim was to identify any significant deviation of premorbid analyte trajectories in those known to develop ALS.

## Materials and methods

### Participants and sampling

#### ALS clinic cohort

Referrals to the Oxford MND Centre clinic between 1 January 2015 and 15 June 2020, aged over 18 years at first clinic visit with a diagnosis of ALS and for whom biomarker data collected through routine healthcare were included. Diagnosis of ALS was made by neurologists experienced in the diagnosis of motor system disorders (K.T., M.R.T. and A.G.T.). Informed consent for wider medical record data analysis received prior ethical committee approval (Health and Social Care Northern Ireland Research Ethics Committee B Reference number 15/NI/0096). Symptom onset was defined as first reported weakness. Historical data for total, HDL and calculated LDL cholesterol, triglycerides, HbA1c (International Federation of Clinical Chemistry units) and creatinine were obtained for patients from Oxford University Hospitals NHS Foundation Trust Department of Clinical Chemistry.

#### UK Biobank cohort

Data were obtained and research conducted through the UK Biobank Resource under application number 57629 (North West—Haydock Research Ethics Committee reference 16/NW/0274). ALS patients were identified using diagnostic codes corresponding to ALS in both UK Biobank inpatient medical record or death certificate linkage (through the UK Biobank algorithmic identification, mapping to International classification of disease (ICD)-10 code G12.2 and ICD-9 code 335.2) and linked primary care data (using read codes mapping to ICD-10 code G12.2 and ICD-9 code 335.2). Date of diagnosis was defined as the earliest ALS code from hospital, primary care or death certificate data. Patients in whom the latency between diagnosis and death or censorship was greater than 10 years were excluded since these are unrepresentative of the majority of ALS cases. Comparator control data were obtained from the UK Biobank primary care record–linked database. Blood test data were extracted from the UK Biobank primary care–linked data by read code. Relevant read codes were identified by searching read code descriptions for a key term (e.g. cholesterol) followed by manual curation, with visual inspection of resulting distributions and exclusion of outliers >5 SDs from the mean following transformation.

One UK Biobank participant without a diagnosis of ALS and at least two measurements of the relevant analyte were matched to each ALS patient by age at first sampling and sex. ALS patients with age of first sampling under 40 years or over 75 years were excluded since there were insufficient data available to enable matching.

### Statistical analysis

Statistical analysis was performed in R. Analyte levels were Box–Cox transformed, with the exception of triglycerides which were log transformed. All transformed analyte levels were mean centred and scaled by standard deviation. Data for ALS patients with at least two measurements of the relevant analyte were analysed. Random intercept and random slope longitudinal mixed effects models of transformed analyte values were fitted, with time measured in relation to onset of first reported weakness in the ALS clinic cohort and in relation to the earliest recorded diagnostic code (by inpatient record linkage or primary care diagnostic read code). Models of triglyceride levels were fitted using random intercept fixed slope models due to singularity when incorporating random slopes.

Inflection points in the data indicating significant deviation from the normal trajectory were sought. Models were created using linear b-splines with zero or one internal knot, allowing the position of internal knots to vary in steps of 3 months. Due to the density of available data, knots were allowed to vary between −8 and +1 year from symptom onset for clinic cohort participants and between −10 and 0 years from diagnosis for UK Biobank ALS patients (−5 and 0 years for HbA1c and −10 and 1 year for creatinine); the model providing the best fit as measured by the Akaike information criterion was selected as the best fitting model for each biomarker, where possible, compared with a model without an inflection point by likelihood ratio test, with the resulting *P*-value adjusted for multiple comparisons using the Bonferroni correction according to the number of possible knot positions. Models were constructed incorporating sex, age of initial sampling and (in UK Biobank participants, for whom prescription data were available) current use of lipid-lowering drugs. Bootstrap confidence intervals (CI) for the inflection point were created using random resampling of data for each participant with 1000 repetitions.

Slope estimates for ALS patient and control data sets and *P*-values for the group: time interactions are given for linear models constructed for timepoints between knots. Parameter estimates presented reflect untransformed values; normalized values in which unity reflects a 1 SD difference from the mean are presented in Supplementary Table 1.

Sensitivity analyses included analysing interactions between age of first sampling and biomarker trajectories, statin use and biomarker trajectories and separately analysing data for exposure to lipid-lowering medication during the period of biomarker analysis (for cholesterol and triglyceride biomarkers).

## Results

Demographic tables for individual analytes are given in Tables 1 and 2. The patient group was slightly older than the mean age of ALS diagnosis in published population-based cohorts, likely due to increasing health screening in older adults and the restricted age of enrolment in the UK Biobank study. In UK Biobank, participants with a diagnosis of ALS also had a higher number of samples taken for LDL cholesterol [ALS patients median 8 (IQR 4–13), controls 5 (3–9), *P* = 0.047] and creatinine [ALS patients 15 (11–25), controls 11 (5–13), *P* = 0.005).

**Table 1:** Demographic information: cholesterol biomarkers

		Total cholesterol				LDL cholesterol				HDL cholesterol		
	ALS clinic	UK Biobank			ALS clinic	UK Biobank			ALS clinic	UK Biobank		
	ALS	ALS	Control	*P*	ALS	ALS	Control	*P*	ALS	ALS	Control	*P*
*n*	86	84	84		60	69	69		86	75	75	
Age at first sampling, median [IQR] (years)	62.60 [54.93–69.25]	57.35 [51.67–60.33]	57.48 [52.13–60.68]	0.838	62.60 [54.55–68.84]	59.47 [54.93–62.14]	58.80 [55.08–61.87]	0.946	62.60 [54.93–69.25]	58.29 [54.17–61.19]	58.63 [53.70–60.83]	0.931
Female, *n* (%)	38 (44.19%)	38 (45.24%)	38 (45.24%)		27 (45.00%)	32 (46.38%)	32 (46.38%)		38 (44.19%)	33 (44.00%)	33 (44.00%)	
Number of samples, median [IQR]	7 [4–12]	11 [6–15]	9 [6–14]	0.423	7 [4–11]	8 [4–13]	5 [3–9]	0.047	7 [4–12]	10 [6–14]	8 [4–13]	0.557
Age of symptom onset or diagnosis, median [IQR] (years)	70.09 [63.23–72.93]	67.81 [63.10–71.05]			70.39 [63.38–74.67]	67.91 [65.12–70.50]			70.09 [63.23–72.93]	67.91 [65.12–70.65]		
Latency from sampling to symptom onset or diagnosis, median [IQR] (years)	−6.64 [−8.59 to −3.32]	−9.01 [−11.71 to −7.59]			−6.91 [−8.71 to −4.87]	−8.38 [−10.05 to −5.17]			−6.64 [−8.59 to −3.32]	−8.70 [−10.77 to −5.19]		
Bulbar onset, *n* (%)	28 (32.56%)				21 (35.00%)				28 (32.56%)			
Survival from symptom onset, median [IQR] (months)	33.22 [28.78–43.99]	21.91 [17.08–26.38]			30.98 [24.71–43.99]	23.03 [17.87–32.92]			33.22 [28.78–43.99]	23.21 [18.53–32.92]		
Genotypes, *n*	*C9orf72*: 2				*C9orf72*: 1				*C9orf72*: 2			

Time anchored to symptom onset in the ALS clinic cohort and diagnosis in the UK Biobank cohort. ALS, amyotrophic lateral sclerosis; HDL, high-density lipoprotein; IQR, interquartile range; LDL, low-density lipoprotein.

**Table 2:** Demographic information: triglyceride, HbA1c and creatinine

		Triglyceride				HbA1c				Creatinine		
	ALS clinic	UK Biobank			ALS clinic	UK Biobank			ALS clinic	UK Biobank		
	ALS	ALS	Control	*P*	ALS	ALS	Control	*P*	ALS	ALS	Control	*P*
*n*	63	76	76		60	23	23		143	103	103	
Age at first sampling, median [IQR] (years)	62.55 [54.28–68.88]	57.86 [54.46–61.15]	58.51 [54.51–60.83]	0.908	63.15 [54.46–69.49]	64.15 [60.48–68.25]	63.33 [60.40–68.41]	0.794	63.41 [54.40–69.70]	58.72 [52.61–62.46]	58.62 [52.92–62.33]	0.978
Female, *n* (%)	28 (44.44%)	34 (44.74%)	34 (44.74%)		25 (41.67%)	8 (34.78%)	8 (34.78%)		66 (46.15%)	48 (46.60%)	48 (46.60%)	
Number of samples, median [IQR]	7 [4–10]	10 [4–14]	8 [5–11]	0.931	10 [3–17]	9 [4–15]	8 [3–12]	0.801	15 [11–25]	11 [7–15]	8 [5–13]	0.005
Age of symptom onset or diagnosis, median [IQR] (years)	70.39 [63.34–74.43]	67.91 [63.90–70.51]			67.27 [58.38–72.18]	67.64 [65.19–70.50]			66.73 [58.41–70.97]	66.91 [61.49–71.05]		
Latency from sampling to symptom onset or diagnosis, median [IQR] (years)	−6.99 [−8.73 to −5.08]	−8.67 [−10.85 to −5.56]			−2.47 [−4.88 to −0.54]	−2.32 [−4.66 to −1.03]			−4.07 [−7.63 to 1.04]	−7.61 [−10.87 to −3.23]		
Bulbar onset, *n* (%)	22 (34.92%)				13 (21.67%)				44 (30.77%)			
Survival from symptom onset, median [IQR] (months)	30.98 [24.71–43.99]	23.03 [17.87–30.92]			43.99 [36.04–NA]	32.03 [14.32–NA]			38.90 [31.51–44.02]	22.87 [18.76–27.07]		
Genotypes, *n*	*C9orf72*: 1								*C9orf72*: 6			

Time anchored to symptom onset in the ALS clinic cohort and diagnosis in the UK Biobank cohort. ALS, amyotrophic lateral sclerosis; HbA1c, glycated haemoglobin A1c; IQR, interquartile range.

Results are summarized in Table 3.

**Table 3:** Summary of results

	ALS clinic cohort						UK Biobank cohort							
	Inflection point		Pre-inflection		Post inflection		Inflection point		Pre-inflection			Post inflection		
	Years from symptom onset	*P*-value	ALS slope	*P*-value	ALS slope	*P*-value	Years from diagnosis	*P*-value	ALS slope	Control slope	ALS versus control *P*-value	ALS slope	Control slope	ALS versus control *P*-value
Total cholesterol (mmol/L/year)	−3.50 [−4.50 to −0.50]	0.008 (0.306)	−0.11	0.001	0.03	0.017	−7.00 [−7.75 to −4.00]	<0.001 (0.009)	−0.25	−0.04	<0.001	0.02	−0.05	<0.001
LDL cholesterol (mmol/L/year)	−1.25 [−3.25 to −0.75]	<0.001 (0.002)	−0.09	<0.01	0.21	0.927	−7.50 [−10.00 to −0.75]	<0.001 (0.005)	−0.24	−0.07	<0.001	0.01	−0.01	0.028
HDL cholesterol (mmol/L/year)	−0.50 [−2.75 to 0.25]	0.004 (0.162)	−0.01	0.163	0.05	0.106	−4.25 [−10.00 to 0.00]	<0.001 (<0.001)	−0.01	−0.01	0.267	0.01	+0.02	0.987
Triglyceride (mmol/L/year)			0.01	0.541					0.00	−0.01	0.480			
HbA1c (mmol/mol/year)	+1.25 [0.00 to +1.50]	0.001 (0.021)	−0.17	0.322	−7.49	0.001	−3.00 [−3.00 to 0.00]	<0.001 (0.001)	+0.92	−3.15	0.561	−0.639	+4.74	0.002
Creatinine (µmol/L/year)	+0.25 [0.00 to +0.50]	<0.001 (<0.001)	−1.81	<0.001	−7.85	<0.001	−0.50 [−1.25 to −0.25]	<0.001 (<0.001)	−1.18	−0.35	0.559	−12.74	+1.35	0.006

*P*-values indicated in brackets are Bonferroni corrected according to the number of possible inflection points. ALS, amyotrophic lateral sclerosis; LDL, low-density lipoprotein; HDL, high-density lipoprotein; HbA1c, glycated haemoglobin A1c.

### Lipids

**Figure 1:**
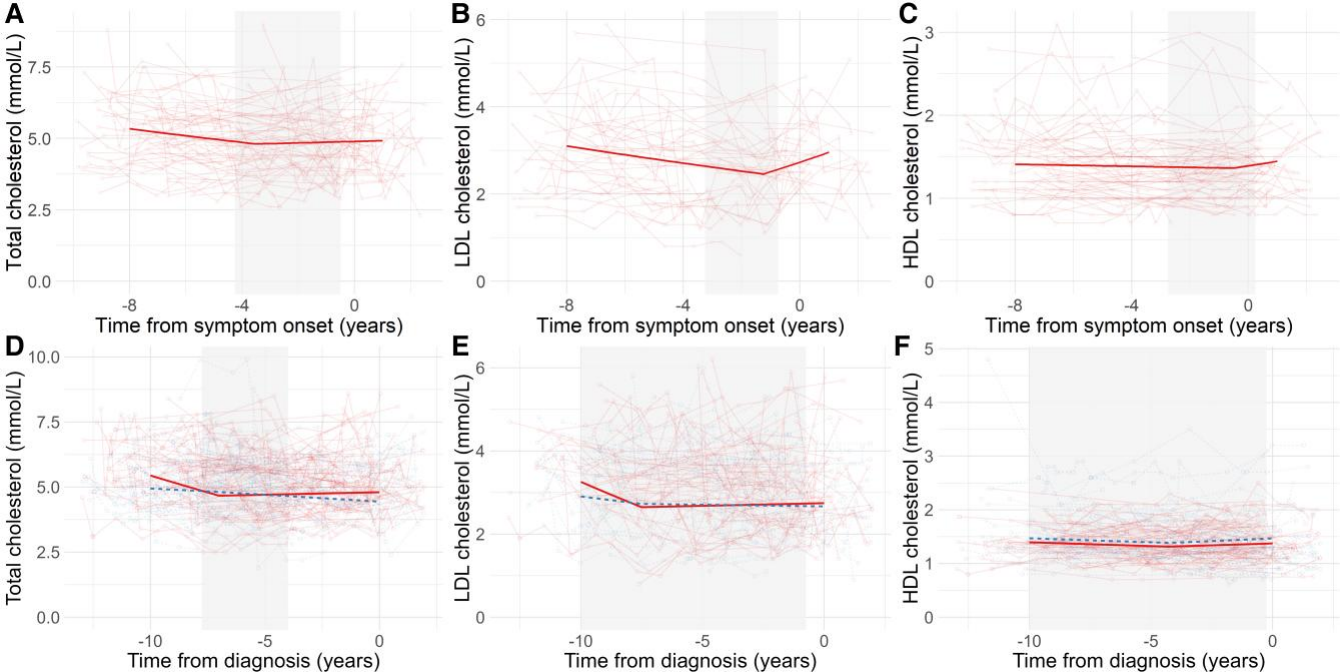
**Temporal changes in cholesterol biomarkers in relation to ALS onset.** Levels of total (**A**, **D**), LDL (**B**, **E**) and HDL (**C**, **F**) cholesterol in relation to symptom onset (ALS clinic cohort; **A**–**E**) or diagnosis (UK Biobank; **D**–**F**). Solid red lines indicate ALS patients, and dashed blue lines age- and sex-matched control participants. Thick line indicates linear mixed models fixed effects for time from symptom onset or diagnosis controlling for sex, use of lipid-lowering drugs and age of first sampling. Shaded area indicates 90% bootstrap CI. ALS, amyotrophic lateral sclerosis; HDL, high-density lipoprotein; LDL, low-density lipoprotein.

Total cholesterol, LDL cholesterol and HDL cholesterol followed an overall similar pattern of change, with consistency between cohorts. Levels of total, LDL and HDL cholesterol declined initially with a nadir prior to symptom onset in the ALS clinic cohort [total cholesterol −3.50 years (90% CI −4.50 to −0.50); LDL cholesterol −1.25 years (−3.25 to −0.75); HDL cholesterol −0.50 years (−2.75 to −0.25)] or diagnosis in the UK Biobank cohort [total cholesterol −7.00 years (−7.75 to −4.00); LDL cholesterol −7.50 years (−10.00 to −0.75); HDL cholesterol −4.25 years (−10.00 to 0.00)], with subsequently stable or rising levels (summarized in Table 3 and Fig. 1).

**Figure 2:**
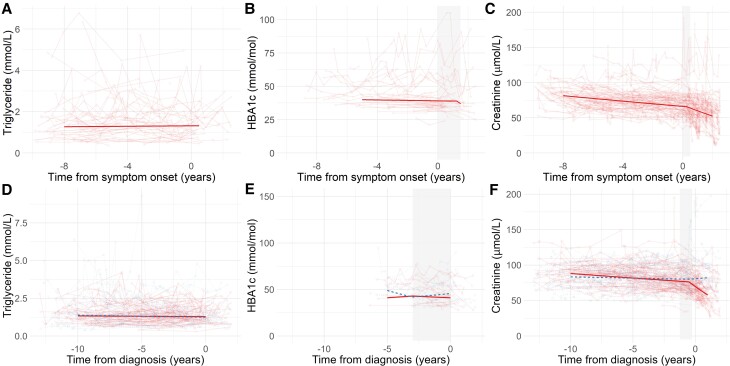
**Temporal changes in triglyceride, HbA1c and creatinine in relation to ALS onset.** Levels of triglyceride (**A**, **D**), HbA1c (**B**, **E**) and creatinine (**C**, **F**) in relation to symptom onset (ALS clinic cohort; **A**–**C**) or diagnosis (UK Biobank; **D–F**). (**F**) Solid red lines indicate ALS patients, and dashed blue lines age- and sex-matched control participants. Thick line indicates linear mixed models fixed effects for time from symptom onset or diagnosis controlling for sex and age of first sampling. Shaded area indicates 90% bootstrap CI. ALS, amyotrophic lateral sclerosis; HbA1c, glycated haemoglobin A1c.

In the UK Biobank cohort, comparisons with control levels indicated that the decline in total and LDL cholesterol prior to diagnosis was more rapid in ALS patients than controls (total cholesterol −0.25 mmol/L/year in ALS patients, −0.04 mmol/L/year in controls, *P* < 0.001; LDL cholesterol −0.24 mmol/L/year in ALS patients, −0.07 mmol/L/year in controls, *P* < 0.001). The subsequent rise in total and LDL cholesterol was more rapid in ALS patients (total cholesterol 0.02 mmol/L/year ALS patients, −0.05 mmol/L/year in controls, *P* < 0.001; LDL cholesterol 0.01 mmol/L/year in ALS patients, −0.01 mmol/L/year in controls, *P* = 0.021). Pre- and post-inflection HDL trajectories did not differ between ALS patients and controls. In contrast, triglyceride levels were constant with no inflection point in both the ALS clinic cohort and UK Biobank cohort and a trajectory that did not differ significantly from controls (Table 3 and Fig. 2).

Trajectories and inflection points were generally robust to sensitivity analyses for total and LDL cholesterol (Supplementary Tables 2–4). Specifically, total cholesterol findings were similar in sensitivity analysis of UK Biobank data including an interaction between age at first sampling and cholesterol trajectory. In the clinic cohort, no inflection point was identified when including an interaction between age at first sampling and cholesterol trajectory (Supplementary Table 2). Similar trajectories and inflection points for LDL cholesterol were identified in sensitivity analyses of both data sets, though due to a small number of patients not exposed to lipid-lowering drugs (*n* = 6), it was not possible to examine this subgroup (Supplementary Table 3). HDL cholesterol findings were not robust in sensitivity analysis (Supplementary Table 4).

### Glycated haemoglobin A1c

Results were inconsistent between the two cohorts. In the ALS clinic cohort, an inflection point was identified 1.25 (0.00 to +1.25) years after symptom onset with levels stable prior to inflection (−0.17 mmol/mol/year, *P* = 0.322) followed by declining levels thereafter (−7.49 mmol/mol/year, *P* = 0.001). In the UK Biobank cohort, an inflection point was identified 3 years (−3.00 to 0.00) years prior to diagnosis, with initially relatively stable levels (+0.92 mmol/mol/year in ALS patients, −3.15 mmol/mol/year in controls, *P* = 0.561) and divergent levels thereafter (−0.630 mmol/mol/year in ALS patients, +4.74 mmol/mol/year in controls, *P* = 0.002; Table 3 and Fig. 2).

### Creatinine

A consistent profile of creatinine levels was observed in both cohorts, with an initial slow decline, followed by a rapid decline in ALS patients. In the ALS clinic cohort, the inflection point was identified +0.25 (0.00 to +0.50) years after symptom onset and in the UK Biobank cohort −0.50 (−1.25 to −0.25) years before diagnosis. The slope prior to the inflection point was similar in ALS patients and controls (−1.18 µmol/L/year in ALS patients, −0.35 µmol/L/year in controls, *P* = 0.559) but after the inflection point, levels declined rapidly in ALS patients (−12.74 µmol/L/year in ALS patients, +1.35 µmol/L/year controls, *P* < 0.001; Fig. 2C). Sensitivity analyses were concordant with the primary analysis (Supplementary Table 5).

## Discussion

This study used blood tests from routine health screening and medical care to consider pre-symptomatic changes in metabolic biomarker levels in those diagnosed with ALS, employing a data-driven approach to identify inflection points in analyte trajectories. There were two main notable findings. Firstly, total and calculated LDL cholesterol showed a biphasic trajectory, which decreased up to an inflection point prior to symptom onset or diagnosis, followed by an upward trajectory. This profile significantly differed from controls in total and LDL cholesterol, and the longitudinal profiles were strikingly consistent between the two cohorts for all cholesterol biomarkers. A similar, but less pronounced, pattern was also observed for HDL cholesterol, though this was not robust to sensitivity analysis. No similar pattern was observed in triglyceride measurements, which remained constant, or in HbA1c, a measure of long-term glycaemic homeostasis.

Differences in the apparent timing are likely attributable to the heterogeneity of the UK Biobank cohort, in particular to the anchoring of disease onset. In the ALS clinic cohort, biomarker sampling is anchored to symptom onset (the most physiologically relevant timepoint), whereas in UK Biobank, this is necessarily anchored to diagnosis obtained from primary care records, hospital inpatient and death certificate linkage. Latency from symptom onset to diagnosis of ALS is known to be highly heterogeneous, with a median latency of 1 year, which may be accentuated in the UK Biobank cohort due to the use of hospital inpatient and death certificate linkage, rather than outpatient encounters where the majority of ALS diagnoses are made. The anchoring of time to diagnosis in UK Biobank and this heterogeneity may also explain the much wider CI for the timing of inflection points in the UK Biobank analysis compared with the ALS clinic cohort. It was not possible to confirm this in the clinic data set since limited data were available for latency to diagnosis. Additional heterogeneity might occur due to erroneous ALS diagnoses in UK Biobank. Analysis of similar data sets indicates a positive predictive value for ALS diagnosis of 70–91%,^24^ though this could not be determined for this specific data set.

Nonetheless, both the ALS clinic and UK Biobank cohorts indicate alterations in the trajectory of cholesterol biomarkers in advance of the emergence of symptoms. In the ALS clinic cohort, this approximately corresponds to the onset of neurodegeneration as measured by neurofilament proteins and neurophysiological testing observed mostly in the year before symptom onset.^4^

Differences in ALS risk in relation to lipid biomarkers have been demonstrated in several large population-based studies, though with a degree of inconsistency, indicating higher risk of ALS in those with higher LDL cholesterol, lower HDL cholesterol and, in one study, higher HDL cholesterol.^10–12^ Our findings suggest that these discrepancies might be attributable to differences in the timing of sampling in relation to ALS symptom onset.

A similar pattern of pre-symptomatic biphasic LDL and HDL cholesterol trajectories in ALS patients, again anchored to diagnosis rather than symptom onset, was described in a large well-controlled longitudinal cohort.^10^ In that study, the apparent nadir of these biphasic responses occurred at ∼10 years prior to diagnosis, substantially earlier than observed in this data set. Differences in the age of the cohorts and the approach to data acquisition are potential contributors to the observed differences. Cross-sectional data from our previous UK Biobank cohort study also provide support for dynamic changes in LDL levels in relation to ALS diagnosis, though it considered only monotonic change.^11^

The reason for this biphasic response is unclear and cannot be delineated from this observational analysis. Cholesterol levels change through adult life, with trajectories that are influenced by genotype.^25^ Around 25% of total body cholesterol resides in the brain, where it is a major constituent of glial and neuronal plasma membranes, particularly myelin.^26^ Plasma cholesterol levels are moderately heritable (around 40% for total, LDL and HDL cholesterol)^27^ and influenced by a range of factors including dietary saturated fats, body fat composition and smoking,^28,29^ which might be altered during subclinical disease accounting for the observed downward trajectory of cholesterol levels. The hypermetabolism that has been associated with symptomatic ALS might also lead to reductions if present during subclinical disease,^30^ contributing to the pre-symptomatic or pre-diagnostic changes observed.

The later rises in cholesterol levels could be attributed to direct or indirect consequences of disease such as muscle loss, reduced activity or dietary modification as part of multidisciplinary care.^31-33^

Exchange between the central nervous system and systemic cholesterol pools is thought to be low (in health), with central nervous system cholesterol largely synthesized *in situ*.^34^ Movement of cholesterol out of the central nervous system has been demonstrated, with central nervous system–derived cholesterol oxides detectable at elevated levels in plasma from people with Alzheimer’s disease.^35^ Elevated cerebrospinal fluid cholesterol levels have been detected in ALS patients,^36^ but given the very low rate of cholesterol flux in the central nervous system, this is less likely to be the cause of increasing levels during the later pre-symptomatic or pre-diagnostic phases demonstrated here.^37^ Cholesterol levels have also been causally associated with ALS through Mendelian randomization studies, and it is possible that cholesterol trajectories might influence risk of ALS or age of onset in susceptible individuals.^13^

The other finding was of longitudinal decreases in plasma creatinine levels, occurring years prior to symptom onset, rapidly accelerating around the time of symptom onset or diagnosis. Analysis including UK Biobank control data suggested that this did not reflect a significant ALS-specific change many years prior to symptom onset but was most likely linked to muscle loss during the symptomatic period. This concurs with a population-based study identifying divergence in creatinine levels in the immediate years before diagnosis of ALS,^20^ though alterations in body mass index as much as 10 years prior to symptom onset have been described.^38^ Creatinine levels are correlated with muscle mass and generally decrease through the disease course.^39^ The finding of sharp falls in creatinine from around the time of symptom onset is consistent with the observation that the major biochemical changes associated with axonal loss, namely neurofilament level rise, occur only months prior to symptom onset in most cases.^4^ This might perhaps support the concept of a ‘tipping point’ in a more long-standing motor system compensation.

There are several limitations that should temper the interpretation of the data presented. Although matched for age and sex, volunteer participants in UK Biobank are known to differ from the general population in terms of lifestyle, ethnicity, health and wealth, which could influence the likelihood of health screening and cardiovascular risk factor modification.^40^ It was not possible to control for changes in body composition and other metabolic measures, such as body mass index, due to a lack of appropriate data within both UK Biobank primary care-linked data sets and ALS clinic cohort data, which could help delineate the cause for the observed temporal patterns.

The analysis of incidentally acquired blood test data may be biased towards older age and co-morbidities with the potential for collider bias. The lipid biomarker conclusions are based on a relatively small number of observations. While this would not be expected to affect the biphasic response, it might impact the accuracy of the timing of inflection points. Finally, clinic attendees were not routinely screened for ALS-causing gene variants until very recently, though the small number of patients with an identified ALS-causing variant did not deviate from the overall trends observed.

Notwithstanding these caveats, the novel data presented here support a growing view that blood lipid level trajectories are significantly altered prior to symptom onset in ALS, perhaps reflecting wider metabolic changes attributable to earlier phases of the neurodegenerative processes. As well as a clue to novel, potentially druggable pathways in ALS pathogenesis, such findings offer the hope of earlier prediction of symptomatic disease onset, though the data presented here only identify trajectory changes at the group rather than the individual level. For those with ALS-causing genetic variants, lipid biomarker trajectories might complement already-identified markers of neurodegeneration in predicting phenoconversion to symptomatic ALS, though further work is required to understand the relevance of these temporal changes at the individual level in this specific group.

## Supplementary Material

fcad211_Supplementary_Data

## Data Availability

The data that support the findings of this study in relation to the ALS clinic cohort are available from the corresponding author, upon reasonable request. UK Biobank is an open-access resource available to verified researchers upon application (http://www.ukbiobank.ac.uk/).
