# Supplementary material for: Primary care blood tests show lipid profile changes in pre-symptomatic amyotrophic lateral sclerosis
Source: Brain Commun. 2023 Jul 28;5(4):fcad211. doi: 10.1093/braincomms/fcad211 (PMC10412752; doi:10.1093/braincomms/fcad211)
Supplement: fcad211_Supplementary_Data [file fcad211_supplementary_data.docx]

**Supplementary material**

**Supplementary table 1** results summary, normalised values. Parentheses indicated Bonferroni corrected p-values. Square brackets indicate 90% confidence interval.

|  | ALS clinic cohort | | | | | |  | UK Biobank cohort | | | | | | | |
| --- | --- | --- | --- | --- | --- | --- | --- | --- | --- | --- | --- | --- | --- | --- | --- |
|  | Inflection point | | Pre-inflection | | Post inflection | |  | Inflection point | | Pre-inflection | | | Post inflection | | |
|  | Years from symptom onset | p-value | ALS slope | p-value | ALS slope | p-value |  | Years from diagnosis | p-value | ALS slope | Control slope | ALS vs control p-value | ALS slope | Control slope | ALS vs control p-value |
| Total cholesterol | -3.50  [-4.50 to -0.50] | 0.008 (0.306) | -0.10 | 0.001 | +0.02 | 0.017 |  | -7.00  [-7.75 to -4.00] | <0.001 (0.009) | -0.22 | -0.04 | <0.001 | +0.02 | -0.05 | <0.001 |
| LDL cholesterol | -1.25  [-3.25 to -0.75] | <0.001 (0.002) | -0.09 | <0.01 | +0.22 | 0.927 |  | -7.50  [-10.00 to -0.75] | <0.001 (0.005) | -0.23 | -0.07 | <0.001 | +0.01 | -0.01 | 0.028 |
| HDL cholesterol | -0.50  [-2.75-0.25] | 0.004 (0.162) | -0.02 | 0.163 | +0.14 | 0.106 |  | -4.25  [-10.00 to 0.00] | <0.001 (<0.001) | -0.04 | -0.04 | 0.267 | +0.04 | +0.05 | 0.987 |
| Triglyceride | - | - | +0.01 | 0.541 | - | - |  | - | - | -0.01 | -0.02 | 0.480 | - | - | - |
| HbA1c | +1.25  [0.00 to +1.50] | 0.001 (0.021) | -0.02 | 0.322 | -0.88 | 0.001 |  | -3.00  [-3.00 to 0.00] | <0.001 (0.001) | +0.09 | -0.30 | 0.561 | -0.06 | +0.11 | 0.002 |
| Creatinine | +0.25  [0.00 to +0.50] | <0.001 (<0.001) | -0.08 | <0.001 | -0.34 | <0.001 |  | -0.50  [-1.25 to -0.25] | <0.001 (<0.001) | -0.06 | -0.02 | 0.559 | -0.67 | +0.06 | 0.006 |

**Supplementary table 2** Total cholesterol sensitivity analyses, non-normalised data. Parentheses indicated Bonferroni corrected p-values. Square brackets indicate 90% confidence interval.

|  | ALS clinic cohort | | | | | |  | UK Biobank cohort | | | | | | | |
| --- | --- | --- | --- | --- | --- | --- | --- | --- | --- | --- | --- | --- | --- | --- | --- |
|  | Inflection point | | Pre-inflection | | Post inflection | |  | Inflection point | | Pre-inflection | | | Post inflection | | |
|  | Years from symptom onset or diagnosis | p-value | ALS slope | p-value | ALS slope | p-value |  | Years from diagnosis | p-value | ALS slope | Control slope | ALS vs control p-value | ALS slope | Control slope | ALS vs control p-value |
| Base model | -3.50  [-4.50 to -0.50] | 0.008 (0.306) | -0.11 | 0.001 | 0.03 | 0.017 |  | -7.00  [-7.75 to -4.00] | <0.001 (0.009) | -0.25 | -0.04 | <0.001 | 0.02 | -0.21 | <0.001 |
| Age of first sampling: time interaction | - | - | -0.03 | 0.023 | - | - |  | -7.00 [-8.00 to -1.00] | <0.001 (<0.001) | -0.27 | -0.05 | <0.001 | 0.02 | -0.21 | 0.246 |
| Anchored to date of diagnosis (n=33) | - | - | -0.02 | 0.427 | - | - |  | - | - | - | - | - | - | - | - |
| Ever statin users (n=76) | - | - | - | - | - | - |  | -7.00 | <0.001 (<0.001) | -0.25 | -0.08 | 0.172 | 0.03 | -0.25 | 0.003 |
| Never statin users (n=8) | - | - | - | - | - | - |  | -4.25 | 0.004 (0.154) | -0.32 | 0.06 | 0.855 | 0.08 | 0.13 | <0.001 |

**Supplementary table 3** LDL cholesterol sensitivity analyses, non-normalised data. Parentheses indicated Bonferroni corrected p-values. Square brackets indicate 90% confidence interval.

|  | ALS clinic cohort | | | | | |  | UK Biobank cohort | | | | | | | |
| --- | --- | --- | --- | --- | --- | --- | --- | --- | --- | --- | --- | --- | --- | --- | --- |
|  | Inflection point | | Pre-inflection | | Post inflection | |  | Inflection point | | Pre-inflection | | | Post inflection | | |
|  | Years from symptom onset or diagnosis | p-value | ALS slope | p-value | ALS slope | p-value |  | Years from diagnosis | p-value | ALS slope | Control slope | ALS vs control p-value | ALS slope | Control slope | ALS vs control p-value |
| Base model | -1.25  [-3.25 to -0.75] | <0.001 (0.002) | -0.09 | 0.001 | 0.21 | 0.927 |  | -7.50  [-10.0 to -0.75] | <0.001 (0.005) | -0.23 | -0.07 | <0.001 | 0.013 | -0.032 | 0.013 |
| Age of first sampling: time interaction | -0.75 [-3.25 to -0.75] | <0.001 (0.005) | -0.08 | 0.410 | 0.33 | 0.166 |  | -7.75 [-10.0 to -0.50] | <0.001 (0.012) | -0.25 | -0.09 | <0.001 | 0.01 | -0.04 | 0.036 |
| Anchored to date of diagnosis (n=22) | - | - | -0.02 | 0.432 | - | - |  | - | - | - | - | - | - | - | - |
| Ever statin users (n=62) | - | - | - | - | - | - |  | -7.75 | <0.001 (0.001) | -0.31 | -0.08 | <0.001 | 0.02 | -0.27 | 0.011 |
| Never statin users (n=7) | - | - | - | - | - | - |  | - | - | 0.09 | -0.27 | 0.093 |  |  |  |

**Supplementary table 4** HDL cholesterol sensitivity analyses, non-normalised data. Parentheses indicated Bonferroni corrected p-values. Square brackets indicate 90% confidence interval. *Identified inflection point not significant after Bonferroni correction.

|  | ALS clinic cohort | | | | | |  | UK Biobank cohort | | | | | | | |
| --- | --- | --- | --- | --- | --- | --- | --- | --- | --- | --- | --- | --- | --- | --- | --- |
|  | Inflection point | | Pre-inflection | | Post inflection | |  | Inflection point | | Pre-inflection | | | Post inflection | | |
|  | Years from symptom onset or diagnosis | p-value | ALS slope | p-value | ALS slope | p-value |  | Years from diagnosis | p-value | ALS slope | Control slope | ALS vs control p-value | ALS slope | Control slope | ALS vs control p-value |
| Base model | -0.50 [-2.75 to 0.25] | 0.004 (0.162) | -0.01 | 0.163 | 0.05 | 0.106 |  | -4.25  [-10.0 to 0.00] | <0.001 (0.009) | -0.014 | -0.014 | 0.017 | 0.014 | 0.079 | 0.522 |
| Age of first sampling: time interaction | - | - | 0.00 | 0.855 | - | - |  | - | - | -0.01 | -0.00 | 0.047 | - | - | - |
| Anchored to date of diagnosis (n=33) | -1.50* | - | 0.05 | 0.098 | - | - |  | - | - | - | - | - | - | - | - |
| Ever statin users (n=68) | - | - | - | - | - | - |  | - | - | -0.00 | 0.01 | 0.135 | - | - | - |
| Never statin users (n=7) | - | - | - | - | - | - |  | -4.75 | 0.001 (0.035) | -0.04 | 0.01 | 0.094 | 0.05 | -0.89 | 0.830 |

**Supplementary table 5** Creatinine sensitivity analyses, non-normalised data. Parentheses indicated Bonferroni corrected p-values. Square brackets indicate 90% confidence interval.

|  | ALS clinic cohort | | | | | |  | UK Biobank cohort | | | | | | | |
| --- | --- | --- | --- | --- | --- | --- | --- | --- | --- | --- | --- | --- | --- | --- | --- |
|  | Inflection point | | Pre-inflection | | Post inflection | |  | Inflection point | | Pre-inflection | | | Post inflection | | |
|  | Years from symptom onset or diagnosis | p-value | ALS slope | p-value | ALS slope | p-value |  | Years from diagnosis | p-value | ALS slope | Control slope | ALS vs control p-value | ALS slope | Control slope | ALS vs control p-value |
| Base model | 0.25 [0.00 to 0.25] | <0.001 (<0.001) | -1.81 | <0.001 | -8.02 | <0.001 |  | -0.50  [-1.25 to -0.25] | <0.001 (<0.001) | -1.18 | -0.34 | 0.559 | -13.35 | 5.40 | 0.006 |
| Age of first sampling: time interaction | 0.25 [0.00 to 0.25] | <0.001 (<0.001) | -1.89 | <0.001 | -6.78 | <0.001 |  | -0.50 [-1.00 to -0.25] | <0.001 (<0.001) | -1.35 | -0.36 | 0.502 | -12.63 | 7.60 | 0.020 |
| Anchored to date of diagnosis (n=46) | 0.00 [-0.5 to 1.75] | <0.001 (<0.001) | -1.22 | 0.037 | -11.34 | <0.001 |  | - | - | - | - | - | - | - | - |
